# The Microscopic Mechanisms of Nonlinear Rectification on Si-MOSFETs Terahertz Detector

**DOI:** 10.3390/s23125367

**Published:** 2023-06-06

**Authors:** Yingdong Wei, Chenyu Yao, Li Han, Libo Zhang, Zhiqingzi Chen, Lin Wang, Wei Lu, Xiaoshuang Chen

**Affiliations:** 1 State Key Laboratory of Infrared Physics, Shanghai Institute of Technical Physics, Chinese Academy of Sciences, 500 Yu Tian Road, Shanghai 200083, China; 2 School of Physical Science and Technology, ShanghaiTech University, Shanghai 201210, China; 3 Hangzhou Institute for Advanced Study, University of Chinese Academy of Sciences, No.1 SubLane Xiangshan, Hangzhou 310024, China

**Keywords:** terahertz (THz) detector, hydrodynamic model, hot-electron effect, non-resonant plasma oscillation regime, nonlinear rectification

## Abstract

Studying the nonlinear photoresponse of different materials, including III-V semiconductors, two-dimensional materials and many others, is attracting burgeoning interest in the terahertz (THz) field. Especially, developing field-effect transistor (FET)-based THz detectors with preferred nonlinear plasma-wave mechanisms in terms of high sensitivity, compactness and low cost is a high priority for advancing performance imaging or communication systems in daily life. However, as THz detectors continue to shrink in size, the impact of the hot-electron effect on device performance is impossible to ignore, and the physical process of THz conversion remains elusive. To reveal the underlying microscopic mechanisms, we have implemented drift-diffusion/hydrodynamic models via a self-consistent finite-element solution to understand the dynamics of carriers at the channel and the device structure dependence. By considering the hot-electron effect and doping dependence in our model, the competitive behavior between the nonlinear rectification and hot electron-induced photothermoelectric effect is clearly presented, and it is found that the optimized source doping concentrations can be utilized to reduce the hot-electron effect on the devices. Our results not only provide guidance for further device optimization but can also be extended to other novel electronic systems for studying THz nonlinear rectification.

## 1. Introduction

The terahertz (THz) wave, ranging from 0.1 to 10 THz, is a region of the electromagnetic spectrum between infrared and millimeter-wave bands and hosts the advantages of optics and microwave electronics [1]. The rising interest in THz technology in fields such as radar, communication, non-destructive detection, biomedical and environmental detection [2,3] has prompted the progressive development of compact THz sources and high-performance detectors.

In the pioneering 1993 theoretical work of Dyakonov and Shur [4,5,6,7,8], they described a comprehensive model for both resonant and non-resonant THz detection and the nonlinear properties of 2D plasma in field-effect transistor (FETs) channels that can be used for detection and mixing of THz radiation. Since their works, a variety of THz detectors made from FETs, such as the silicon metal-oxide semiconductor field-effect transistors (Si-MOSFETs) [9,10,11,12], high-electron-mobility transistors (HEMTs) [13,14,15,16,17,18,19], graphene field-effect transistors [20,21,22], organic–inorganic perovskite photodetectors [23,24] and newly emerging topological materials, have been widely explored. For III–V materials, such as GaN/AlGaN HEMT hosts high carrier mobility and 2D electron gas density induced by the spontaneous/piezoelectric polarization effect of the wurtzite material, showing high responsivity and low noise equivalent power (NEP) at room temperature [25]. In the meantime, other heterostructures such as GaAs/AlGaAs, InGaP/InGaAs/GaAs and InGaAs/AlInAs have also been proposed as active THz detectors with fast response and a high signal-to-noise ratio [26,27].

Recently, there have been a number of positive developments in international research around the optoelectronic properties of electronic systems in low-dimensional materials. As the most classical two-dimensional material, it has shown new vitality in a series of important applications such as ultra-broadband, high-speed optical communication and biosensing, which overturn traditional functions [28]. Meanwhile, other low-dimensional materials, such as graphene [29,30,31], black phosphorus [32,33,34] and transition metal dichalcogenides (TMDCs) [35,36,37,38,39], all exhibit extremely strong nonlinear THz rectification effects. Many efforts have been made to realize the modulation of the structural and electronic properties of low-dimensional semimetal materials by optical techniques, such as high-order harmonic generation [40] and light frequency mixing [41]. These works open new possibilities for the development of dissipation-less and ultrafast topological devices in data processing, sensing, and communication [42]. However, the performance of THz photodetectors is limited due to the low photon energy of the THz band, weak light absorption, and low collection efficiency of carriers [43]. In addition, conventional THz photodetectors are also limited by low speed, low operation temperature, and high power requirements. Therefore, it is challenging to realize room-temperature high-performance THz photodetectors [44,45]. To overcome these difficulties, we have explored the physical process of nonlinear THz rectification based on a typical material— silicon. It will be of great help for further research on future THz nonlinear rectification mechanisms based on graphene, black phosphorus, topological Dirac/Weyl semimetals and others.

In this work, we use a finite element method [46,47] to better present the physical picture of nonlinear rectification via a self-consistent solution of transport equations related to different material parameters. Different harmonics are coupled since the nonlinear equations are solved. Extensive time-domain simulations are performed to uncover the carrier dynamics in the channel following the hydrodynamic and drift-diffusion models. Typically, the non-resonant THz detection of the device with different structural parameters and different doping concentrations has been discussed. Our results not only provide guidance for further device optimization but can also be extended to other novel electronic systems for studying THz nonlinear rectification, which will be very useful for future research into low-energy photon harvesting techniques.

## 2. Concept of Nonlinear Rectification in a Quasi-One-Dimensional Analysis Model

Harmonic generation is a general characteristic of nonlinear systems; the process of nonlinear rectification in MOSFETs for THz radiation also exhibits this feature [4,5,6,7,8,40]. Following Dyakonov and Shur’s work, the nonlinear properties of plasma waves due to the oscillation of carrier density and velocity play a central role in THz radiation rectification [4,5,6,7,8]. The 2D electron gas will exhibit interesting hydrodynamic behavior [3,4], and the steady current flow in a FET channel can lead to the growth of plasma waves [4]. The AC voltage generated by the incident THz light will act on the channel of the FET, and the nonlinear current–voltage dependence will generate different orders of signal due to the harmonic generation [42,43,44]. Thus, the physical process of THz nonlinear rectification is described as follows.

The surface concentration *n* in the MOSFET channel is related to the local gate-to-channel voltage swing above the threshold and can be simply determined by the gradual channel approximation [4,5,6,7,8]:

(1) n=CU/q

where *C* is the gate-to-channel capacitance per unit area; *q* is the electron charge and *U* = *U_gc_(x)* − *U_T_*; *U_gc_* is the gate-to-channel voltage; and *U_T_* is the threshold voltage.

The hydrodynamic Euler equation and current continuity equation can be used to understand the dynamic processes of carriers according to the theory of Dyakonov and Shur [4,5,6,7,8], which can be written as follows:

(2) $$\frac{\partial v}{\partial t}+v\frac{\partial v}{\partial x}+\frac{q}{m}\frac{\partial U}{\partial x}+\frac{v}{\tau }=0$$

(3) $$\frac{\partial n}{\partial t}-\frac{1}{q}\frac{\partial j_{n}}{\partial x}=0$$

where *v* is the local electron velocity, ∂*U*/∂*x* is the longitudinal electric field in the channel, *m* is the effective electron mass, τ is the momentum relaxation time, *n* is the electron concentration, and *j_n_* is the electron current density (for the n-type channel).

Considering Equation (1), the usual continuity equation should be used to calculate Equation (2), which is as follows:

(4) $$\frac{\partial U}{\partial t}+\frac{\partial \left(U\nu \right)}{\partial x}=0$$

These equations coincide with hydrodynamic equations for shallow water. The growth of plasma waves caused by current flow in the FET channel is similar to the shallow water wave [4,5,6,7,8].

Besides, the boundary conditions are as follows:

(5) $$U\left(0,t\right)=V_{gs}+U_{a}\sin\left(\omega t\right),\;for\;x=0$$

(6) $$j\left(L,t\right)=0,\;\;for\;x=L$$

where *V_gs_* is the dc gate-to-source voltage swing, *U_a_* is the amplitude representing the intensity of the THz radiation, and *ω* is the angular frequency of the incident THz radiation. $U_{ac}=V_{gs}+U_{a}\sin\left(\omega t\right)$ is the external AC voltage induced between the gate and source by the incoming THz wave, and j=CUν is the electron current per unit width.

Then, the solution of Equations (2) and (4) is described in the following form:

(7) $$\nu =\overset{-}{\nu }+\nu_{1}+\nu_{2}\ldots$$

(8) $$U=\overset{-}{U}+U_{1}+U_{2}\ldots$$

where $\overset{-}{\nu },\overset{-}{U}$ are the time-averaged electron velocity and channel potential, respectively, and $\nu_{n},U_{n}$ vary with time with the frequency nω, where ω is the frequency of the input signal. Since Equations (1) and (2) are nonlinear equations, different harmonics are coupled. It is worth noting that the nonlinear properties of plasma waves can provide the possibility of optical detection [4,5,6,7,8]. However, if the input signal, *U_a_*, is relatively small, $\nu_{1},U_{1}$, are proportional to *U_a_*, while $\overset{-}{U}-U_{0},\overset{-}{\nu }$, and $\nu_{2},U_{2}$, are proportional to *U_a_*^2^, etc. Because the incident THz electromagnetic waves are small in energy, *U_a_* is a small quantity, and *U_a_*^2^ is an even smaller magnitude. Finally, the embodied higher-order harmonics are dominated by the second-order harmonics.

To the first order in *U_a_*, the following equations are obtained:

(9) $$\frac{\partial v_{1}}{\partial t}+\frac{\partial u_{1}}{\partial x}+\frac{v_{1}}{\tau }=0$$

(10) $$\frac{\partial u_{1}}{\partial t}+s^{2}\frac{\partial v_{1}}{\partial x}=0$$

where *u*_1_ = *eU*_1_/*m* and *s* = *(eU*_0_*/m)*^1/2^ is the plasma wave velocity. Retaining the second-order time-independent terms, we find the following:

(11) $$\frac{d}{dx}\left(\overset{-}{u}+\frac{\langle \nu_{1}^{2}\rangle }{2}\right)+\frac{\overset{-}{\nu }}{\tau }=0$$

(12) $$\frac{d}{dx}(s^{2}\overset{-}{v}+\langle u_{1}\nu_{1}\rangle )=0$$

Here, $\overset{-}{u}=e\overset{-}{U}/m$ and the angular brackets denote the time averaging over the period 2π/ω. The boundary conditions for (9)–(12) follow from (5) and (6):

(13) $$u_{1}\left(0\right)=\frac{e}{m}U_{a}cos\omega t$$

(14) $$\overset{-}{u}\left(0\right)=\frac{e}{m}U_{0}$$

(15) $$\overset{-}{v}\left(L\right)=v_{1}\left(L\right)=0$$

For the boundary conditions given by (14) and (15), the integration of (11) and (12) with respect to x yields the following:

(16) $$\Delta u=\overset{-}{u}\left(L\right)-\overset{-}{u}\left(0\right)=\frac{1}{2}\langle v_{1}^{2}\left(0\right)\rangle -\frac{1}{\tau }\int_{0}^{L}\overset{-}{v}dx$$

(17) $$\overset{-}{v}=-\frac{\langle u_{1}v_{1}\rangle }{s^{2}}$$

Furthermore, the evaluation of Δu from solving the plasma wave dispersion equation, yields the detector response ΔU=mΔu/e, which is the constant source-to-drain voltage induced by the incoming ac signal [4,5,6,7,8].

(18) $$\frac{\Delta U}{V_{gs}}=\frac{1}{4}{\left(\frac{U_{a}}{U_{0}}\right)}^{2}f\left(\omega \right)$$

where

(19) $$f\left(\omega \right)=1+\frac{2\omega \tau }{\sqrt{1+{\left(\omega \tau \right)}^{2}}}$$

Depending on the collision-induced damping of the plasma wave oscillations in the channel, the detection mode is composed of resonant and non-resonant modes, which are determined by the excitation frequency *ω* and the momentum relaxation time *τ*.

When ωτ≫1, the detector works as a resonant detector. The damping of the plasma oscillations is small under the illumination of incident light. The plasma waves reflect between the two boundaries of the channel, causing continuous plasma wave oscillation. The resonance occurs at a specific frequency $\omega_{N}=\omega_{0}(2N+1)$ leading to a sharp resonance, $\omega_{0}=\pi s/2L_{g}$, *s* is the plasma wave velocity and *L_g_* is the channel length [4,5,6,7,8,24,25].

On the other hand, the detector works in the nonresonant mode due to the overdamping of the plasma wave when ωτ≪1. In this case, the DC voltage between the source and drain is a constant. Compared with the case in the non-resonant mode, the generated photoresponse signal could be improved several times in the resonant mode [4,5,6,7,8]. However, several factors, such as channel length and phonons or impurity scattering processes, could limit the signal, which is usually prominent at room temperature. Therefore, we only focus on the THz detector operating in the non-resonant mode at room temperature [4,5,6,7,8].

Theoretically, Equations (1) and (2) indicate the physics of 2D electrons and plasma waves in a simple form. Nonlinear properties of the 2D electron gas lead not only to the rectification of the incoming electromagnetic radiation but also to the emergence of the signal with the second-order and higher harmonics of the incoming radiation [4,5,6,7,8]. It can be seen from Equation (19) that the photoresponse changes only by a factor of three, even when the parameter ωτ increases from zero to a very high value. Even though the underlying physical process may be different, the voltage-tunability of FETs endows them with unique advantages to reach higher performance.

## 3. Simulation Model and Equations

Based upon the simplest description of nonlinear rectification in one-dimensional terms, we would like to introduce more intricate situations of nonequilibrium dynamics when considering realistic devices. In this work, Sentaurus Technology Computer-Aided Design (TCAD L-2016.03-SP2) simulation is explored via the hydrodynamic/drift-diffusion transport model with extensive incorporation of different physical quantities, open circuit boundary conditions, etc., which describes the plasma wave in the MOSFET channel in a self-consistent manner [4,48,49,50]. The drift-diffusion transport model is used when in thermodynamic equilibrium [50]. When further considering the lattice and electron temperatures, we will use the hydrodynamic model included in the software for the simulation [51,52,53]. During the simulation process, the drift-diffusion model, the Mobility model, the Hydrodynamic model and the RSH (Shockley–Read–Hall) model contained in the simulator are fully used. Here, the basic equations used in the Sentaurus Device are described as follows.

Firstly, the software solves the Poisson equation, which is described as follows:

(20) $$\nabla^{2}\phi =\frac{q}{\varepsilon }\left(N+p-n\right)$$

where, ϕ is the electrostatic potential, *q* is the elementary electronic charge, ε is the permittivity, *n* is the electron density, and *p* is the hole density.

Secondly, the software solves the continuity equation. In addition, in this device, the charge carriers are electrons, which is described as follows:

(21) $$\nabla \cdot \vec{J_{n}}=qR_{net,n}+q\frac{\partial n}{\partial t}$$

where, $R_{net,n}$ is the electron net recombination rate, $\vec{J_{n}}$ is electron current density.

Thirdly, the software solves the drift-diffusion transport equation, which is described as follows:

(22) $$\vec{J_{n}}=-nq\mu_{n}\left(\nabla \varphi_{n}\right)$$

where, $\varphi_{n}$ is the Quasi-fermi potential and $\mu_{n}$ is the electron mobility. During the simulation, the drift-diffusion transport equations can, eventually, be extended to the hydrodynamic equations.

Fourthly, the thermodynamic transport equation differs from drift-diffusion when the lattice temperature equation is solved. However, it is possible to solve the lattice temperature equation even when using the drift-diffusion model.

(23) $$\vec{J_{n}}=-nq\mu_{n}\left(\nabla \varphi_{n}+p_{n}\nabla T\right)$$

where, $p_{n}$ is the absolute thermoelectric power and *T* is the lattice temperature.

Fifthly, when the electron temperature is also considered in the hydrodynamic model in TCAD, the solution equation is the following:

(24) $$\vec{J_{n}}=\mu_{n}(n\nabla E_{c}+kT_{n}\nabla n-nkT_{n}\nabla \ln\gamma_{n}+\lambda_{n}f_{n}^{td}k_{n}\nabla T_{n}-1.5nkT_{n}\nabla \ln m_{n})$$

in Equation (24), the first term takes into account the contribution due to the spatial variations of electrostatic potential, electron affinity, and the band gap. The remaining terms in Equation (24) take into account the contribution due to the gradient of concentration, the carrier temperature, eventually *T_e_* in the channel, gradients, and the spatial variation of the effective masse, *m_n_*. for Boltzmann statistics, $\gamma_{n}=\lambda_{n}=1$. The thermal diffusion constant $f_{n}^{td}$ defaults to zero, which corresponds to the Stratton model in TCAD [51,52]. Theoretically, the carriers absorb photon energy via an interband or intraband transition under radiation, which leads to the average temperature *T_e_* rising above the lattice temperature *T*. This transport model of Equation (24) takes into account the non-equilibrium state between hot carriers and lattice temperature for different channel sizes, which can be used to study the microphysical behavior of hot electrons [51,52,53].

By self-consistently solving the above physical equations, it allows for the description of the microphysical processes at the interior of the device and ensures the accuracy of the simulation results. It will also provide a convenient way to study the nonlinear rectification process in two-dimensional materials, which opens the door for modification of physical and material parameters [29,30,31,32,33,34,35,36,37,38,39].

## 4. Simulation Procedure for Finite Element Analysis

Finite Element Analysis (FEA), by using the numerical calculation method, is an efficient tool to solve all kinds of scientific problems [46,47]. Many problems can be solved by using the finite element method, including electromagnetics, heat transfer, fluid mechanics, stress analysis and other problems [54,55,56]. Meanwhile, it is widely used in scientific fields such as aerospace, automotive, electronics, nuclear science and other fields [50,51]. To date, more and more complex problems in the field of science need to be extracted from their physical models and even consider the interaction of various physical fields. At this moment, FEA can be used to combine a variety of complex physical models and solve complex scientific problems [57,58,59].

The finite element method is used by partitioning a continuous entity into multiple subdomains [54]. In this way, the entity is partitioned into a discrete body with no intersection between each subdomain within the body. The discretized subdomains after partitioning are used as differential elements representing the entity model [60,61]. Separate analysis of each differential element is achieved by solving differential equations created for each element and analyzing the solution results of the differential equations [62,63,64].

The physical field equations that satisfy the behavior of carriers in semiconductors [47] can be solved in TCAD, such as voltage and current magnitude, electron-hole movement and recombination [65,66]. The device is divided into numerous subdomains, and the finite element analysis is performed in each subdomain. Each subdomain of the device is interlinked, such as the contact area between the adjacent subdomains, the type of material added, and the doping concentration of the added material [67,68,69]. The specific simulation steps are described as follows.

**Figure 1:**
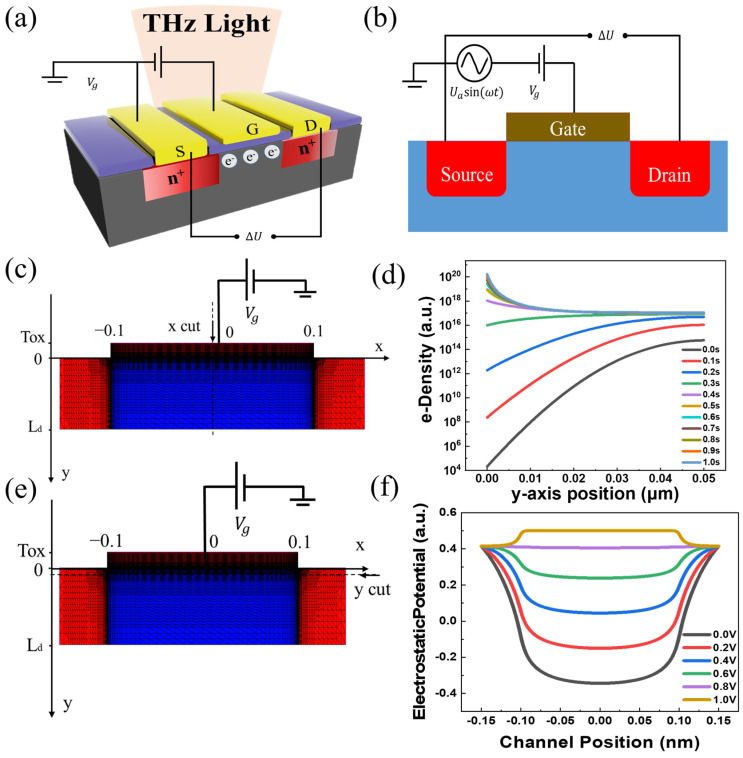
(**a**) MOSFET THz detector structure. (**b**) Circuit schematic for dynamic simulation. (**c**,**d**) Electron concentration of the channel at x = 0 as the gate voltage increases linearly. (**e**,**f**) Surface electrostatic potential over the range of gate voltage from 0 to 1 V.

The schematic of the silicon-based FETs is shown in Figure 1a. The length of drain *L_d_* and source *L_s_* is 50 nm, the channel length *L_g_* is 100–300 nm, and the ${SiO}_{2}$ insulating layer is 10 nm. Figure 1b shows the main parameters used in the simulation. The dc gate voltage *V_gs_* and AC voltage *U_a_sin(ωt)* are provided between the gate and source (the source terminal is grounded), and *V_ds_* is measured with an open circuit boundary condition. Figure 1c shows the finite element division of the mesh structure. Within a specified period of time, the electron concentration of the channel at x = 0 as the gate voltage linearly increases from 0 to 1 V is shown in Figure 1d. During the gate voltage scanning, electron concentration increased by 16 orders of magnitude. Figure 1e,f show the surface electrostatic potential over the range of gate voltage from 0 to 1 V. These figures show that the channel potential continues to rise, and eventually an antipattern layer is formed. Figure 1e shows the cross-section of the channel at y = 3 nm. Figure 1f shows that the surface electrostatic potential rises, extending along the x-axis plane in the channel, as the gate voltage scans. Theoretically, it indicates that the channel is conducting, which can be further used for the internal physical process of the detector. Next, Table 1 lists the important parameters for silicon materials [54].

**Figure 2:**
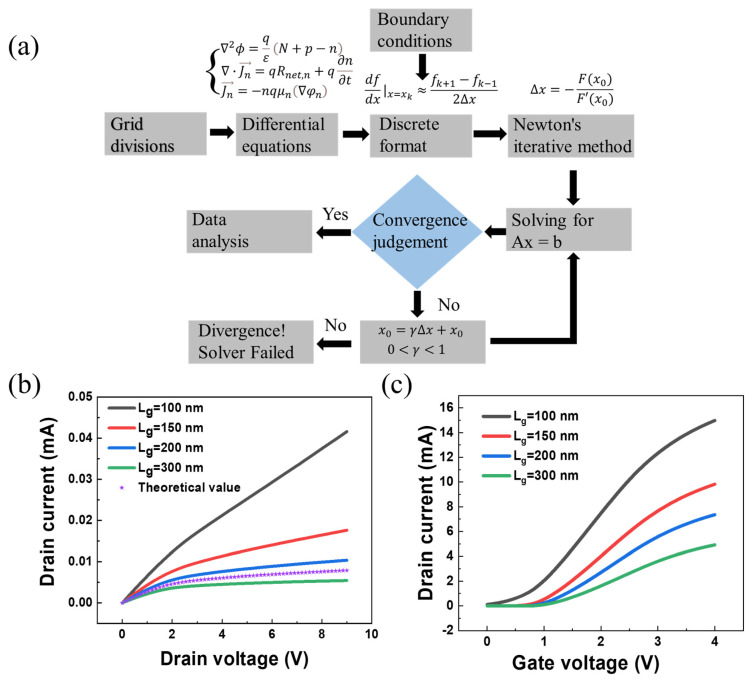
(**a**) Flow chart of finite element simulation. (**b**) Output characteristics of MOSFETs with different channel lengths. (**c**) Transfer characteristic curves of devices with different channel lengths.

Figure 2a illustrates the process of finite element simulation and summarizes the main flow of the device simulation. The device is divided into finite grids, and the equations are solved in each grid considering the boundary conditions. The solution results are output after convergence judgment. Figure 2b shows the output current–voltage characteristics of the MOSFET simulated at the gate voltage *V_g_* = 3 V and different gate lengths. Meanwhile, the simulation results have the same trend as the previously calculated theoretical results, which further validates that our finite element simulation is reliable [70]. Figure 2c shows the transfer current–voltage characteristics of the transistor calculated at the drain voltage *V_d_* = 2 V. One can find that the device threshold voltage (*V_th_*) increases as the *L_g_* increases. Specifically, when the *L_g_* is 300 nm, the *V_th_* is 1.23 V, which is significantly larger than that of 100 nm as attributable to a larger parasitic capacitance.

Next, detailed quasi-continuous time-domain simulations are performed to analyze THz mixing in the MOSFET channel. Following Dyakonov and Shur’s work, the nonlinear properties of the 2D electron gas led to THz rectification and the appearance of the signal at the second and higher harmonics under THz radiation [4,5,6,7,8].

**Figure 3:**
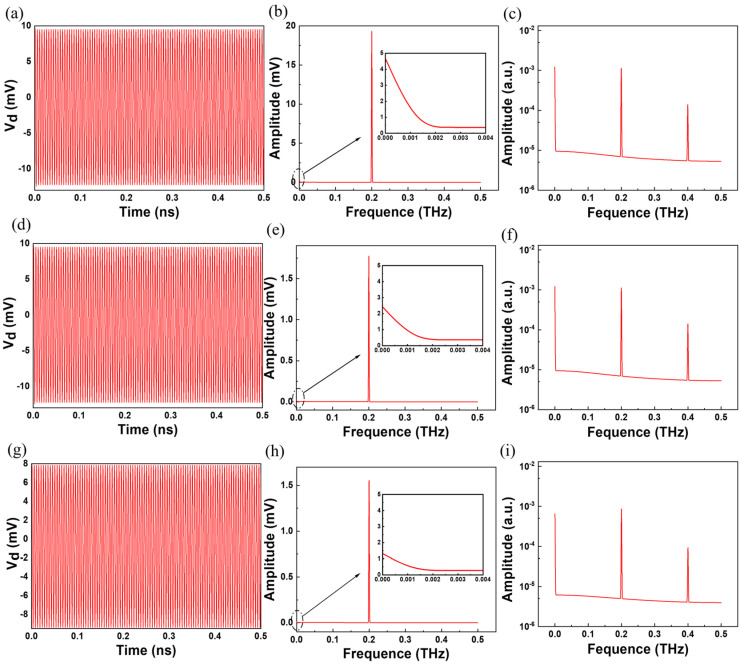
With *V_gs_* at 0.7 V, (**a**) Time-domain simulation for *V_ds_*. (**b**) Fourier transform for the response with amplitude at 0.2 THz. (**c**) FFT was plotted in the logarithmic coordinate system. With *V_gs_* at 0.8 V, (**d**) Time-domain simulation for *V_ds_*. (**e**) Fourier transform for the response with amplitude at 0.2 THz. (**f**) FFT was plotted in the logarithmic coordinate system. With *V_gs_* at 0.9 V, (**g**) Time-domain simulation for *V_ds_*. (**h**) Fourier transform for the response with amplitude at 0.2 THz. (**i**) FFT was plotted in the logarithmic coordinate system.

Figure 3a shows the time evolution of *V_ds_* at the gate bias of 0.7 V with a channel length of 100 nm. It is worth noting that the drain output is comprised of harmonics and self-mixing DC components. To extract the dc response voltage, we performed a Fourier transform to obtain the frequency domain characteristics of the output signal amplitude (Figure 3b). It can be observed that a fundamental tone of *ω* = 0.2 THz (the incident frequency) is accompanied by a distinguishable DC component (Figure 3b, in the inset). Moreover, as shown in Figure 3c, the second harmonic (0.4 THz) can be observed. Figure 3d–f show the optical response of the device at *V_gs_* = 0.8 V. Figure 3g–i shows the optical response of the device at *V_gs_* = 0.9 V.

In general, the fundamental harmonics (0.2 THz) and the second harmonics (0.4 THz) are in along with the DC component (*f*~0). As a detector, the DC component can be extracted as the photovoltaic response and is generally used as a signal for THz detection.

**Figure 4:**
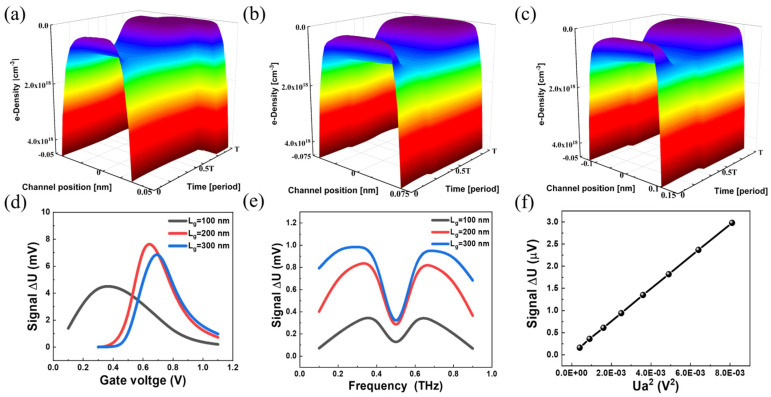
Plot of electron concentration with period and channel position at (**a**) 100, (**b**) 200 and (**c**) 300 nm for asymmetric boundary conditions. (**d**) Optical response of the device at different gate voltages. (**e**) Optical response of the device under different THz radiation with frequency changed from 0.1 to 0.9 THz range, different channel length device optical response, and the gate voltage fixed at threshold voltage. (**f**) Square-law dependence of response on RF signal amplitude at *V_gs_* = 0.7 V.

By using the hydrodynamic model (corresponding to the drift-diffusion model in software), the open circuit boundary conditions, we simulate the incoming THz wave as a sinusoidal AC signal of frequency (ω), which induces 2D electrons and plasma waves to oscillate in the channel. Here, the oscillations can be captured in a two-dimensional distribution after a rigorous calculation of the transport equation. With the open circuit boundary condition, Figure 4a–c show the simulated results of 2D electron and plasma wave oscillation as a function of time and position along the channel, corresponding to the *L_g_* of 100, 200 and 300 nm.

In agreement with Equations (2), (4) and (18), which describe the plasma wave oscillation with an open circuit boundary condition, we can see a significant asymmetric shift in the electron concentration within one oscillation cycle. The asymmetric shift is caused by the interplay between nonlinear electron oscillation and asymmetric boundary conditions along the channel, leading to a change in potential distribution after time accumulation. Besides, it can be found that the asymmetric offset becomes more pronounced as the *L_g_* increases, which is probably caused by the loading effect reported before [71,72,73,74,75].

Figure 4d shows the drain output direct current (DC) voltage signal Δ*U* with the change of gate-to-source static voltage for different channel lengths *L*_g_. With the increase in *L_g_*, Δ*U* initially increases, and then it can be seen that Δ*U* reaches its peak at *L_g_* = 200 nm. At channels longer than 200 nm, the signal starts to decrease as a result of the loading effect from the access region in the MOSFET device, and the efficiency is reduced at the expense of sub-threshold swing.

Besides, in Figure 4e, we plot the optical response of devices with different *L_g_* from 0.1 to 0.9 THz. It can be observed that the response reaches its lowest value at 0.5 THz. At *L_g_* = 100 nm, the response of the device is smaller than in the other two cases. However, such a process is inefficient at higher frequencies due to the adverse effects of parasitic capacitance and resistance [70]. By taking into account the level of response and reliability, it can be concluded that the optimal length is at *L_g_* = 200 nm.

As a detector, the linear dynamic response is another important parameter that describes the power-dependent properties of targeting weak objects. A good linear relationship between DC output and normalized input radiation power ($U_{a}^{2}$) obtained by changing the power by more than an order of magnitude, is shown in Figure 4f [40]. Indeed, it can be inferred from Figure 2, Figure 3 and Figure 4 that the simulated results display very good output and transfer characteristics, and plasma-mixing with high efficiency is given rise at the drain-end under THz irradiation and asymmetric open-circuit boundary conditions. These results validate the reliability and feasibility of our approach for designing and optimizing such types of devices, according to the literature [53].

Following the results of Figure 2c and Figure 4d, an interesting phenomenon can be identified: the *V_th_* is minimal when the *L_g_* is 100 nm, and the saturation curve bends significantly upward as *V_d_* increases. In Figure 4d, the response signal does not grow monotonously after increasing both *V_g_* and *L_g_* and reaches its peak near the threshold gate voltage at *L*_g_ = 200 nm. Such a trend cannot be described by simply following Equation (18), from which only a constant voltage drop can be derived, manifesting the discrepancy of gradual channel approximation from the more realistic case that our numerical modeling captures, which means that the physical processes are more complicated when the device is operated below the threshold voltage *V_th_*. Furthermore, our results are also in good agreement with other experimental results for gate voltage-dependent properties, which all show the maximum response near *V_th_* [76,77,78].

## 5. Impact of Hot Electrons on Optical Detection

In order to understand the role of non-equilibrium hot electronic processes, we have investigated the physical behavior of carriers in MOSFET short-channel structures. When the channel length of the device is less than the thermal relaxation length of the material, the hot electron effect cannot be neglected in the case of detectors [79].

In 2014, Wang et al. first revealed the physical mechanism of photocurrent generation inside the detector, confirming that THz irradiation can generate a current from hot electrons [80,81]. Under the low energy of terahertz irradiation, it forms hot carriers in the channel with an average temperature *T_e_* above the lattice temperature *T* [82,83]. Theoretically, the formation of hot carriers is much faster than the rate of thermal equilibrium with the lattice, and the hot carrier temperature can remain above the lattice temperature for a considerable period of time [79,82,83]. As a result, it creates a temperature gradient Δ*T* in the channel. During this period, some of the hot carriers in the channel spontaneously diffused from the high-temperature region to the low-temperature region under the drive of the temperature gradient, which generates current in the channel [79,82,83]. For our simulated process, the hot carriers are hot electrons, whose free diffusion leads to hot electron effects that result in a reduced optical response for THz nonlinear rectification. Theoretically, the ability of hot carrier diffusion is expressed by the Seebeck coefficient, and the potential built into the channel under THz radiation is that ΔU=−SΔT [79]. The direction of the resulting current depends on the variation of the material Seebeck system, which is non-monotonic under THz irradiation [80,81,82,83].

The following figure plots the distribution of different electron temperatures during one THz radiation oscillation cycle. In the hydrodynamic model, we have considered the impact of temperature induced by hot electrons on the current. Moreover, other variables are kept constant unless specifically stated.

**Figure 5:**
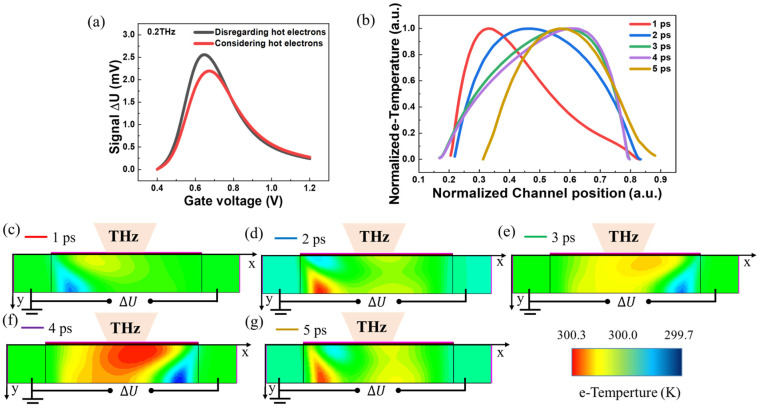
(**a**) Hot-electron effect on the optical response of the device. (**b**) Distribution of different electron temperatures during a 0.2 THz radiation oscillation cycle. (**c**–**g**) Two-dimensional distribution of different electron temperatures within one cycle of THz radiation oscillations.

Figure 5a shows that the optical response is somewhat reduced when considering the physical processes in which hot electrons are present. Therefore, we further analyze the internal variation of the detector during a 0.2 THz oscillation cycle. The electron temperature distribution at different times within a THz oscillation cycle (from 0 to 5 ps) is shown in Figure 5c–g. One can find that the highest temperature point of the electron gradually moves from the source to the drain and finally returns to its original state. We further extract the one-dimensional electron temperature profiles along the horizontal direction of the device (Figure 5b), which intuitively illustrates the movement of the electron temperature peak. In this case, a temperature gradient in the channel is formed due to the non-uniform temperature distribution, which results in the directional diffusion of hot electrons from the drain to the source and a reduction of the non-linear terahertz response at the drain output.

Following the previous device simulations, our finite-element model can be further verified by varying the source/drain doping concentrations and examining the hot electron effect on the output and transfer characteristic curves of the device [84].

**Figure 6:**
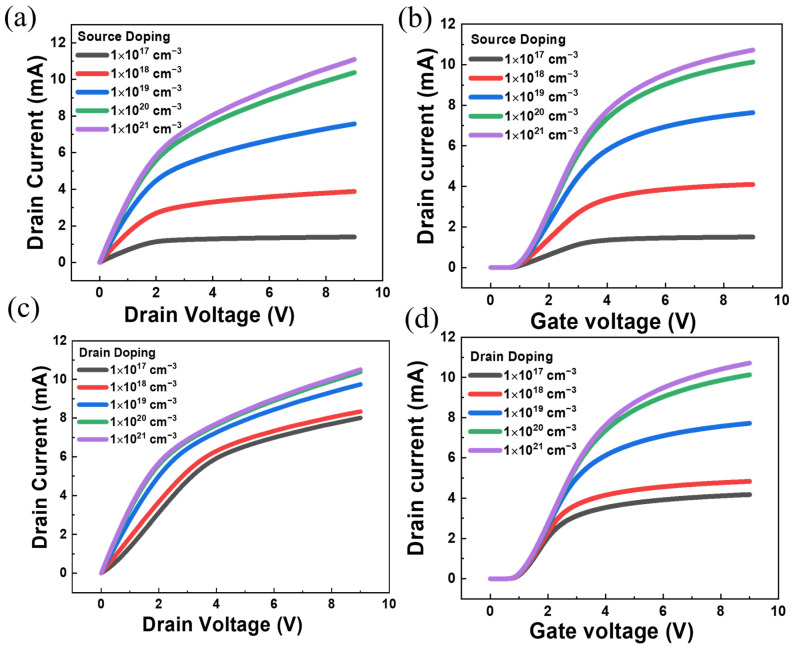
(**a**) I-V curves of devices with increasing doping concentrations at the source with a 200 nm channel. (**b**) Transfer characteristics curves with increasing doping concentrations at the source. (**c**) I-V curves of devices with increasing doping concentration at drain with a 200 nm channel. (**d**) Transfer characteristics curves with increasing doping concentrations at the drain with a 200 nm channel.

Figure 6a–d display the transfer and output characteristic curves by changing the doping at the source and drain, respectively. At first, we calculate the *V_th_* of the transfer curve at different doping concentrations, and *V_th_* is 1.05 V at drain doping of 10^17^ cm^−3^, *V_th_* is 1.05 V at drain doping of 10^18^ cm^−3^, *V_th_* is 1.07 V at drain doping of 10^19^ cm^−3^, *V_th_* is 1.11 V at drain doping of 10^20^ cm^−3^, and *V_th_* is 1.12 V at drain doping of 10^21^ cm^−3^. The doping concentrations in the range of 10^18^ to 10^20^ cm^−3^ show an obvious effect on the transferring curve, while little effect is observed with doping below or beyond this regime.

Next, we would like to reveal the effect of doping at the source on the device’s performance. The *V_th_* is growing up by increasing the concentration, e.g., *V_th_* is 0.87 V at source doping 10^17^ cm^−3^, *V_th_* is 0.97 V at source doping 10^18^ cm^−3^, *V_th_* is 1.07 V at source doping 10^19^ cm^−3^, *V_th_* is 1.11 V at source doping 10^20^ cm^−3^, and *V_th_* is 1.12 V at source doping 10^21^ cm^−3^. The output characteristic curves exhibit a saturated trend at intermediate doping concentrations between 10^17^ and 10^20^ cm^−3^. This can reduce the effect of hot electrons on the output characteristics of the device.

**Figure 7:**
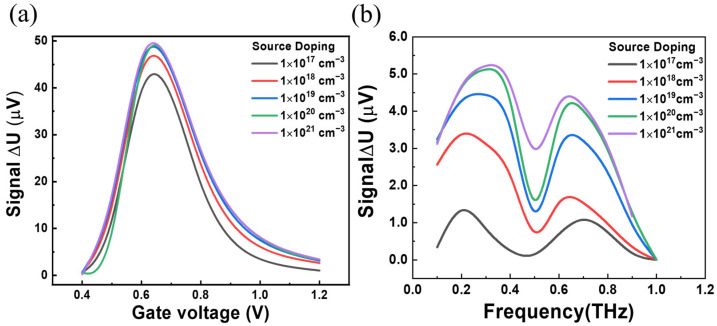
(**a**) Optical response of 200 nm channel devices doped with different source concentrations at different gate voltages. (**b**) Optical response of 200 nm channel devices doped with different source concentrations at a 0.7 V gate voltage in the frequency range of 0.1 to 0.9 THz.

Following the above results, the response curves of the device under THz irradiation by changing the doping at the source side are simulated and shown in Figure 7. Under different gate voltage modulations, it can be clearly seen that the device hosts a good response when the doping is between 10^19^ and 10^20^ cm^−3^ in Figure 7a. Furthermore, the highest value of the device response shifts toward a larger *V_g_* as the doping concentration increases. When the doping concentration is below or above the range from 10^18^ to 10^20^ cm^−3^. Based on this, the optimum source doping concentration can be selected.

Furthermore, the optical responses related to different source doping concentrations and different frequencies from 0.1 to 1.0 THz are presented in Figure 7b. It can be found that the optimal doping concentration is between 10^19^ and 10^21^ cm^−3^, and the response remains high with bandwidth exceeding 0.65 THz, which may help in other novel materials for studying THz nonlinear rectification.

## 6. Conclusions

In this work, we have demonstrated the physical mechanism of nonlinear THz rectification by a finite element method by self-consistently solving the transport equation dynamically. Extensive time-domain simulations are performed to show the carrier dynamics in the channel, considering the hydrodynamic and drift-diffusion models. The competitive behavior between nonlinear rectification and hot electrons induced by the photothermoelectric effect is clearly presented in nanoscale detectors. It is found that the hot-electron effect can be reduced by optimizing the source doping concentrations. Our results provide opportunities for device optimization and understanding the nonlinear THz rectification in other electronic systems, enabling low-energy photon harvesting techniques.

## Figures and Tables

**Table 1:** Physical Properties of Silicon.

Properties	Si
Effective density of states in the conduction band, *N_c_* (cm^−3^)	$2.8\times 10^{19}$
Effective density of states in the valence band, *N_v_* (cm^−3^)	$1.04\times 10^{19}$
Electron affinity (V)	4.05
Energy gap at 300 K (eV)	1.12
Minority carrier lifetime (s)	$2.5\times 10^{-3}$
Electron Mobility (drift) (cm^2^/V s)	1500
Hole Mobility (drift) (cm^2^/V s)	450

## Data Availability

The datasets used and/or analyzed during the current study are available from the corresponding author on reasonable request.
